# Mitochondrial Diabetes Mellitus With Mitochondrial DNA 3316G>A Mutation: A Unique Autopsy Case Presenting With Sepsis-Associated Cholestasis

**DOI:** 10.7759/cureus.57418

**Published:** 2024-04-01

**Authors:** Chikara Mashiba, Akihiro Shioya, Motona Kumagai, Mitsuteru Yoshida, Sohsuke Yamada

**Affiliations:** 1 Department of Pathology, Kanazawa Medical University Hospital, Uchinada, JPN; 2 Department of Pathology and Laboratory Medicine, Kanazawa Medical University, Uchinada, JPN; 3 Department of Pathology II, Kanazawa Medical University, Uchinada, JPN

**Keywords:** sepsis-associated cholestasis, gram-negative bacteremia, conjugated hyperbilirubinemia, mitochondrial dna 3316g>a mutation, mitochondrial diabetes mellitus (mdm)

## Abstract

A 70-year-old female, diagnosed with mitochondrial diabetes mellitus (MDM) showing previously a point mutation at mitochondrial DNA 3316G>A, noticed urinary tract infection and diabetic gangrene of the foot with Gram-negative *Bacteroides fragilis* bacteremia, followed by aggressive jaundice with high serum level of direct bilirubin. She died two months after the symptom onset. At autopsy, multiple foci of bacteremia-induced hemorrhagic infarction were observed in the congestive bilateral lungs, whereas the cholestatic liver revealed no overt gross cholangiectasis. Microscopic findings characteristically showed many bile thrombi in the biliary canaliculi of hepatic lobules without any evidence of severe shock liver. Finally, we diagnosed it exclusively as sepsis-associated cholestasis due to the marked elevation of Gram-negative bacteria-derived endotoxins and inflammatory cytokines. We propose that these unique liver features in our MDM case might be one of the new clues to unveil its enigmatic etiology.

## Introduction

Mitochondrial diseases are known to be maternally inherited disorders characterized by mitochondrial DNA mutations, manifesting as various and heterogeneous clinicopathological features, including sensorineural deafness or diabetic nephropathy [1]. Although phenotypic variability should be due to the homoplasmy or the degree of heteroplasmy for the point mutation in different tissues with different thresholds, mitochondrial genes are well known to be candidates for diabetes mellitus (DM) [2,3]. Mitochondrial diseases account for only 0.01%-0.02% of people [1], whereas the accurate frequency of mitochondrial diabetes mellitus (MDM) among those patients remains unclear. However, according to the recent retrospective analysis of a Japanese cohort study, the prevalence of MDM is estimated to be around 0.08% among patients who had kidney biopsies [4]. It has been reported that the point mutation at mitochondrial DNA 3243A>G is closely correlated with MDM and occurs in up to 6% of patients with end-stage renal failure associated with DM [5]. Furthermore, based on the Japanese studies’ results, the point mutation at mitochondrial DNA 3316G>A seems to be also frequent, accounting for approximately 3% and 1% in randomly selected DM and control subjects, respectively [6]. However, only a few cases with detailed clinicopathological examination were reported [7], and there have been no autopsy papers regarding MDM with mitochondrial DNA 3316G>A mutation, within our thorough investigation.

We describe a unique autopsy case of MDM with mitochondrial DNA 3316G>A mutation, in which a characteristic sepsis-associated cholestasis was finally recognized during the final pathological examinations, and the clinical diagnosis of the cause of aggressive jaundice with conjugated hyperbilirubinemia was very difficult.

This article was previously posted to the medRxiv preprint server on May 18, 2023.

## Case presentation

A 74-year-old female was diagnosed with MDM showing a G to A transition at the nucleotide pair 3316 of mitochondrial DNA by the direct nucleotide sequence at the age of 38 [7]. The proband was a nondrinker and nonsmoker, and she had a history of diabetic triopathy and sensorineural deafness since about 50 years of age. Regarding the proband’s family history, the same homoplasmic mutations as mitochondrial DNA 3316G>A were noted in the peripheral leucocytes of her mother and twin sons [7], and her sons died with mitochondrial cardiomyopathy-induced myocardial infarction or end-stage renal failure on the age of 44 and 30. She had been treated with hemodialysis due to diabetic nephropathy and end-stage renal failure since the age of 66. On the current admission, she had just noticed a urinary tract infection and diabetic gangrene of the foot with blood culture-positive *Bacteroides fragilis* (Gram-negative) bacteremia two months before her death, followed by aggressive jaundice with conjugated hyperbilirubinemia for the last 27 days. A physical examination revealed the following: height, 151 cm; body weight, 45.8 kg; and body mass index (BMI), 20.1. A subsequent brain/chest/abdominal CT scan revealed multiple thrombi in the cerebrum, lungs, and spleen and one right renal mass, but the liver showed no significant change.

The patient’s laboratory studies (one day before her death) displayed obstructive jaundice and severe infection (Table 1). However, the status of the serum levels of inflammatory cytokines, such as tumor necrosis factor-alpha (TNF-α) or interleukin 1 beta (IL-1β), was not available.

**Table 1:** The patient’s laboratory studies (one day before her death).

Laboratory parameters (with units)	Patient values	Reference range
Aspartate aminotransferase (AST) (U/L)	30	13-30
Alanine aminotransferase (ALT) (U/L)	7	7.0-23
Alkaline phosphatase (ALP) (U/L)	1,016	38-113
Gamma-glutamyl transpeptidase (GGTP) (U/L)	76	9-32
Lactate dehydrogenase (LD) (U/L)	246	124-222
Prothrombin time-international normalized ratio (PT-INR)	1.33	0.9-1.1
Fibrinogen (mg/dL)	186	150-350
Fibrin degradation product (FDP) (μg/mL)	34.5	0-5
D-dimer (μg/mL)	19.4	0-1
Total bilirubin (mg/dL)	12.2	0.4-1.5
Direct bilirubin (mg/dL)	10.0	0-0.4
C-reactive protein (CRP) (mg/dL)	16.93	0-0.14
White blood cell (WBC) count (/mm^3^)	9,330	3,300-8,600
Hemoglobin (Hb) (g/dL)	8.8	11.6-14.8
Blood urea nitrogen (BUN) (mg/dL)	82	8.0-20
Creatinine (mg/dL)	7.01	0.46-0.79

An arterial blood gas (ABG) analysis revealed the following: pH, 7.421; partial pressure of carbon dioxide (PaCO_2_), 28.5 mmHg; partial pressure of oxygen (PaO_2_), 80.5 mmHg; and bicarbonate (HCO_3_-), 18.5 mEq/L. For the clinicians, it was very difficult to explain the cause of aggressive jaundice with high serum levels of direct bilirubin. She died of multiple organ failure, especially liver failure, 27 days after this symptom onset.

**Figure 1:**
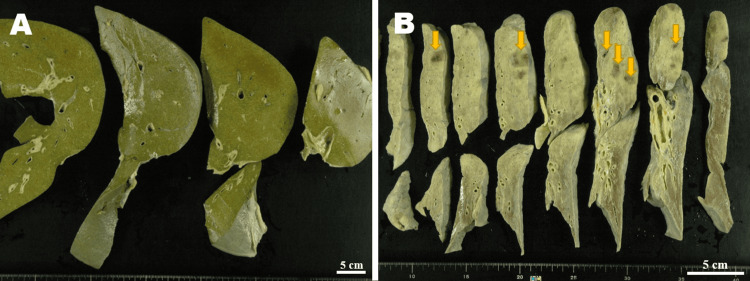
The autopsy gross findings of the liver and lung. (A) The cholestatic liver was green in color on the cut surface; however, no overt cholangiectasis was recognized. The background of the liver was not apparently ischemic or cirrhotic. (B) On the cut surface of the congestive left lung, multiple foci of bacteremia-induced hemorrhagic infarction (arrows) were observed especially in the upper lobe.

At autopsy, the cholestatic liver, weighing 910 g, was grossly green on the cut surface; however, no overt cholangiectasis was identified (Figure 1A). The background of the liver was not apparently ischemic or cirrhotic. The severely atrophic left kidney (45 g) showed infection-induced pyuria and hydronephrosis, and the atrophic right kidney (145 g) contained a mass tumor lesion (diameter: >6 cm). In addition, multiple foci of bacteremia-induced hemorrhagic infarction were observed especially in the upper lobes of the congestive bilateral lungs (weighing 370 g {left} and 300 g {right}) (Figure 1B). The enlarged heart, weighing 345 g, showed apparent concentric ventricular hypertrophy and old myocardial infarction in the left ventricle. The other organs had no significant change. The brain could not be examined due to the family’s objections.

**Figure 2:**
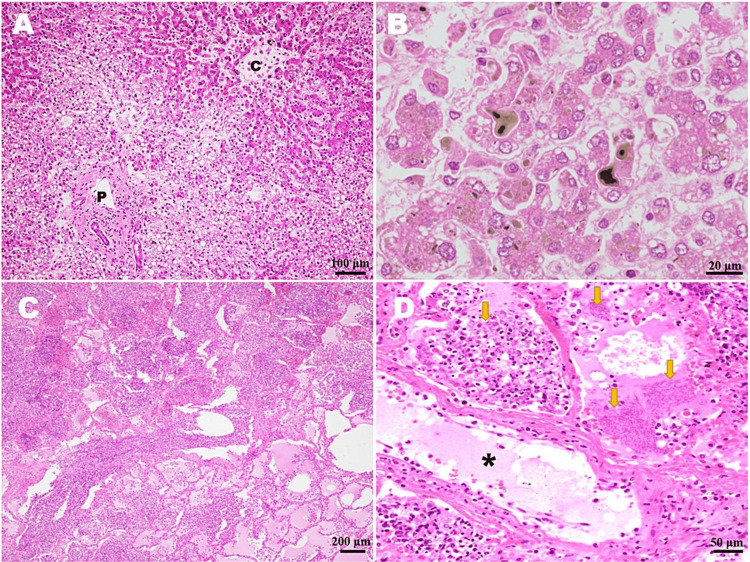
The autopsy histological findings of the liver and lung. (A) In the cholestatic liver, the portal area showed no remarkable change, and there were no apparent intrahepatic biliary stones. As to the hepatic lobules, there was no definite evidence of severe shock liver or acute inflammation, and foci of septic micro-abscess were not seen (C, central vein; P, portal area). (B) On a high-power view of hepatic lobules, many bile thrombi in the biliary canaliculi were characteristically identified. The atrophic hepatic trabeculae contained small lipid droplets. (C) The edematous lung contained foci of hemorrhagic infarction with acute inflammation. (D) On a high-power view of lung infarction, many colonies of Gram-negative rod-shaped bacilli (arrows) were readily recognized. Also, frequent vascular endothelial denudation (asterisk) was associated with neutrophilic attachment and infiltration.

Histologically, the liver characteristically showed many bile thrombi in the biliary canaliculi of the hepatic lobules without any evidence of severe shock liver or acute inflammation (Figure 2A, 2B). Foci of septic micro-abscess were not identified. The portal area had no remarkable change, and there were no apparent intrahepatic biliary stones (Figure 2A). The atrophic hepatic trabeculae contained small lipid droplets in part (Figure 2B). The edematous lungs showed multiple hemorrhagic infarctions with many colonies of Gram-negative rod-shaped bacilli (Figure 2C, 2D). On a high-power view of bacteremia-induced pulmonary infarction, vascular endothelial denudation associated with neutrophilic attachment and infiltration was readily seen (Figure 2D).

**Figure 3:**
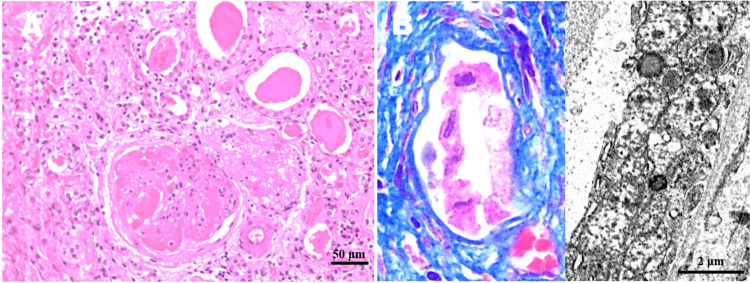
The histological/ultrastructural findings of the kidney. (A) On a high-power view of the atrophic left kidney, Kimmelstiel-Wilson nodules were identified as a diabetic nephropathy, in the background of arteriolosclerosis and pyelonephritis. (B) Masson’s trichrome staining revealed a characteristic swollen change in the epithelia of the kidney collecting tubule (left: Masson’s trichrome staining) (original magnification: ×400). Moreover, on electron microscopy, an ultrastructural analysis of the uriniferous tubule showed an increased number of abnormal swollen mitochondria varying in size and shape with loss of cristae (right).

Microscopically, the atrophic and diabetic kidneys contained one right typical clear cell renal cell carcinoma, in the background of acute on chronic pyelonephritis, diabetic nephropathy, and atherosclerotic change (Figure 3A). Kimmelstiel-Wilson nodules were occasionally seen, whereas no overt focal segmental glomerulosclerosis was noted. Masson’s trichrome staining very rarely revealed a characteristic swollen change in the epithelia of kidney collecting tubules (Figure 3B). Moreover, on electron microscopy, an ultrastructural analysis of the uriniferous tubule displayed an increased number of abnormal swollen mitochondria varying in size and shape with loss of cristae (Figure 3B). Finally, the pancreatic islets microscopically and immunohistochemically demonstrated that β cells were obviously decreased in less than approximately 30% associated with focal deposits of amorphous material, amyloid.

Based on all these features, we have concluded that the compromised state of the present MDM patient with mitochondrial DNA 3316G>A mutation caused severe urinary tract infection and diabetic gangrene of the foot with *Bacteroides fragilis* bacteremia. The bacteremia-induced sepsis resulted in multiple organ failure, including renal, respiratory, and liver failure, at the very least. With regard to the liver failure displaying aggressive jaundice with conjugated hyperbilirubinemia, we finally diagnosed it exclusively and conclusively as sepsis-associated cholestasis due to the marked elevation of Gram-negative bacteria-derived endotoxins and inflammatory cytokines.

## Discussion

Although jaundice with hyperbilirubinemia is a frequent symptom in the intensive care unit with an incidence of up to 40% among critically ill patients [8,9], sepsis-associated cholestasis could not be a common feature for both clinicians and pathologists. However, it has been described that cholestasis is a possible complication in septic patients with extrahepatic Gram-negative bacterial infection [10,11], as in the present autopsy case. We should be aware that, in particular, septic patients with aggressive jaundice and conjugated hyperbilirubinemia undergo a fatal condition with a very poor prognosis [11]. Actually, few case reports and review articles available on cholestasis in sepsis [8-11] have suggested that a confident and conclusive diagnosis might be impossible based on a clinical examination alone. Therefore, it is likely that sepsis-associated cholestasis is frequently overlooked by not only pathologists but also clinicians, and it must be more common than generally considered. According to a recent review paper, cholestasis in sepsis results from (i) harmful effects of Gram-negative bacteria-derived endotoxins and inflammatory cytokines on the downregulated expressions of various important proteins, including hepatocellular transporters of bile acids and bilirubin, and (ii) following impairments in bile secretion and/or bile flow at the level of small or large bile ducts due to the additional hepatic ischemia and hypoperfusion [10,11]. The correct diagnosis of sepsis-associated cholestasis might require an accurate approach for an adequate biopsy specimen from the liver, after distinguishing this established entity from other cholestatic diseases, such as obstructing bile stones, severe ischemic/hypoxic liver injury (i.e., shock liver), or progressive sclerosing cholangitis [8-11]. In the current case, we were able to easily rule out those possibilities, since the liver histology showed no significant change in the portal and lobular areas, except for the characteristic of many bile thrombi in the biliary canaliculi, as shown in Figure 2A/Figure 2B.

In patients of mitochondrial diseases, especially MDM, it is well known that mitochondrial dysfunction through the disordered mitochondrial oxidative phosphorylation should lead to the development of DM associated with decreased insulin secretion via the functional alterations in adenosine triphosphate (ATP) generation [7,12]. In fact, it has been reported that the nucleotide pair 3316 in the mitochondrial DNA belongs to the nicotinamide adenine dinucleotide hydrogen (NADH) dehydrogenase subunit 1 (ND1)-encoding region, which critically constitutes the mitochondrial oxidative phosphorylation system [13]. Also, the mitochondrial DNA 3316G>A mutation is known to be closely correlated with not only MDM but also Leber’s hereditary optic neuropathy or Alzheimer’s disease [7], even though the phenotypic variability of mitochondrial diseases should be due to the homoplasmy or the degree of heteroplasmy for the point mutation in different tissues with different thresholds at least in part [2,3]. Here, we speculate that the patient with mitochondrial DNA 3316G>A mutation might be linked to high susceptibility to sepsis-associated cholestasis. Actually, with regard to MDM showing a point mutation of mitochondrial DNA 3316G>A, very few cases with detailed clinicopathological findings have been published, and there are no autopsy reports. We hypothesize that mitochondrial DNA 3316G>A mutation might be able to play a crucial role in the pathogenesis of sepsis-associated cholestasis, via reducing mitochondrial ATP and increasing the generation of mitochondrial oxidative stress, potentially associated with following increased intrahepatic proinflammatory cytokines and apoptosis. Nevertheless, the present unique liver features in our MDM case might be one of the new clues to unveil its enigmatic etiology. Further prospective studies are needed to validate the presence and significance of sepsis-associated cholestasis, after collecting and investigating a larger number of MDM cases with mitochondrial DNA 3316G>A mutation examined.

## Conclusions

In conclusion, we herein report an autopsy case of MDM with mitochondrial DNA 3316G>A mutation, in which characteristic sepsis-associated cholestasis was exclusively and conclusively diagnosed by the pathological examinations, and it was very difficult for clinicians to explore the cause of aggressive jaundice with conjugated hyperbilirubinemia. Although sepsis-associated cholestasis is not a common feature, we should be aware that its correct diagnosis might require an approach for an adequate biopsy specimen from the liver, after distinguishing it clearly from other cholestatic diseases.
